# Correlation of clinicopathological and prognostic characteristics between endometriosis-associated and primary ovarian cancer

**DOI:** 10.1186/s12885-023-11641-4

**Published:** 2023-12-08

**Authors:** Huimin Wang, Cong Chen, Danbo Wang, Yanmei Zhu, Peng Chen

**Affiliations:** 1 Department of Gynecology, Liaoning Cancer Hospital & Institute, 44 Xiaoheyan Road, Dadong District, Shenyang, Liaoning 110004 China; 2 Department of Pathology, Liaoning Cancer Hospital & Institute, Shenyang, Liaoning China

**Keywords:** Endometriosis-associated ovarian cancer, Ovarian clear cell carcinoma, Ovarian endometrioid carcinoma, Diagnostic criteria

## Abstract

**Background:**

The main aim of this study was to establish the clinicopathological and prognostic correlations between endometriosis-associated and non-endometriosis-associated primary ovarian cancer, with a view to providing a reference guide for revision of diagnostic criteria for malignant transformation of endometriosis.

**Methods:**

Clinicopathological and follow-up data of 174 patients with clear cell and endometrial ovarian cancer were retrospectively extracted. Cases were divided into endometriosis-associated and non-endometriosis-associated primary ovarian cancer for comparative analysis of clinicopathological characteristics and prognosis.

**Results:**

Average age and post-menopausal rate in the endometriosis-associated ovarian cancer group were lower relative to the primary ovarian cancer group (*P* < 0.05). Body mass index, age at menopause, operation history, dysmenorrhea, complications, tumor size, tumor side, ascites, CA125, HE4, CA19.9, stage, differentiation, expression of ER, PR, P53, P16, Ki67, MMR, HNF-1β and Napsin A were not significantly different between the groups (*P* > 0.05). Furthermore, rates of resistance to platinum chemotherapy, relapse, progression-free survival and overall survival were comparable between the two groups (*P* > 0.05).

**Conclusion:**

Endometriosis-associated and primary ovarian cancers of the same pathological type are speculated to be homologous in terms of origin from malignant transformation of endometriosis. It may therefore be necessary to revise the diagnostic criteria for ovarian endometriosis malignancy.

## Background

Endometriosis is a common disease in women of childbearing age, with an incidence of 15–20% [1]. Studies to date have shown that endometriosis has the same molecular biological and genetic background as ovarian cancer and presents a high risk factor for ovarian cancer development [2]. According to the current internationally recognized Sampson and Scott diagnostic criteria: (1) there must be a clear example of endometriosis in association with or close proximity to the cancer, (2) no other primary tumor site must exist and the histology of the tumor should be consistent with an endometrial origin, (3) endometriosis associated with cancers must show morphologic progression from benign to malignant in a contiguous fashion [3, 4], with a malignant transformation rate of 0.5–1.0% [5]. However, due to the significant heterogeneity of endometriosis and atypical hyperplasia, high rates of missed pathological diagnosis are inevitable and the actual incidence of malignant transformation of endometriosis may be higher [6, 7], which presents a challenge in establishing accurate diagnostic criteria.

The major pathological types of endometriosis-associated ovarian cancer are endometrioid and clear cell cancer, accounting for 75–90% cases [8]. However, according to the diagnostic criteria of Sampson and Scott, only 50–70% ovarian clear cell and endometrioid carcinoma cases are diagnosed as endometriosis-associated ovarian cancer (EAOC). Based on the theory of external origin of ovarian cancer, clear cell and endometrioid carcinoma types are closely related to endometriosis, potentially resulting from endometriosis-associated malignancy [9]. Our research is focused on the malignant transformation of endometriosis. Previous studies have reported comparable abnormal expression of EAOC-related genes, such as mismatch repair gene human mutL homolog 1 (hLMH1) and runt-related transcription factor3 (RUNX3), between EAOC and non-endometriosis-associated primary ovarian cancer (non-EAOC) groups [10, 11], giving rise to the speculation that all clear cell and endometrioid carcinomas of the ovary originate from malignant transformation of endometriosis. Since the pathological changes of endometriosis are heterogeneous, particularly after malignant transformation, tumor tissues grow vigorously and destroy the original tissues, and the histological basis of endometriosis thus remains unclear. Pathological sampling has a number of limitations. For instance, cancer and endometriosis lesions are not obtained at the same time and concurrent endometriosis is often overlooked, resulting in a low diagnostic rate of malignant transformation of endometriosis using the standard criteria of Sampson and Scott. Further relevant clinical and basic research is thus warranted to validate this theory.

## Materials and methods

### Research objects

In total, 174 cases of ovarian clear cell carcinoma and ovarian endometrioid carcinoma were diagnosed and treated in China Medical University Cancer Hospital and Affiliated Shengjing Hospital from January 2008 to November 2018. Participants were divided into EAOC (74 patients) and non-EAOC (100 patients) groups according to the standards of Sampson and Scott, as follows: (1) coexistence of cancer and endometriosis in the same ovary, (2) a similar histological pattern, (3) exclusion of secondary tumors metastatic to the ovary, and (4) histopathological evidence demonstrating the transition from benign endometriosis to malignancy. Patients that met the above criteria were classified as EAOC and the remaining patients as non-EAOC. According to the different pathological types, endometriosis-associated ovarian clear cell carcinoma (EOCC) and endometriosis-associated ovarian endometrioid carcinoma (EOEC) groups were further defined. Non-endometrium-related primary ovarian cancer was further grouped into clear cell carcinoma (OCC) and endometrioid carcinoma (OEC) groups. Our patient population included 39 EOCC, 35 EOECC, 35 OCC and 65 OECC cases. Samples were fully encoded to protect patient confidentiality.

Inclusion criteria were as follows: (1) definitive pathological diagnosis, limited to clear cell carcinoma and endometrioid carcinoma, (2) complete case data, and (3) completion of the initial treatment plan (surgery and chemotherapy). Exclusion criteria were as follows: (1) the presence of other histological types of ovarian malignancies and borderline tumors of non-clear cell carcinoma and endometrioid carcinoma, (2) other primary malignant tumors, and (3) metastatic ovarian cancer. Samples of the selected cases were re-assessed by the same gynecological pathologist to further confirm diagnosis.

### Data collection

Clinicopathological data of all patients were collected. Clinical parameters included age, body mass index (BMI), dysmenorrhea or not, menopausal status, age of menopause, endometriosis history, operation or not (for instance, cesarean section, hysterectomy, endometriosis), complications (for instance, diabetes, heart disease, high blood pressure), related serum tumor markers (CA125, CA19.9, HE4), type of surgery (complete, suboptimal, and optimal), and endometriosis lesions during surgery. Pathological parameters included tumor size, tumor side, ascites, FIGO stage, histological classification, and immunohistochemical results (ER, PR, P53, P16, Ki67, MMR, HNF-1, Napsin A).

### Treatments

All the included patients received standard surgical treatment. Patients in the early stage (FIGO I-II) received comprehensive staging surgery while those in the advanced stage (FIGO III-IV) received tumor cell reduction surgery. Postoperative chemotherapy regimens containing platinum were adopted according to international guidelines as follows: paclitaxel and carboplatin (TC regimen), docetaxel and carboplatin (DC regimen), 6–8 cycles of chemotherapy, with an interval of 21 days.

### Follow-up

All cases were followed up until the end of recurrence, death or the end of follow-up (up to 31/3/2019). The follow-up time was 6-132 months, with median of 67 months. Cases requiring reoperation to obtain histopathological and/or imaging evidence of new developments and/or continued abnormal elevation of tumor markers were considered tumor recurrence.

### Statistical analysis

Data were analyzed using SPSS 25.0 software. Enumeration data are expressed in terms of rates and measurement data as mean ± standard deviation (± sd). Student’s T test was used for comparison between groups. The enumeration data were compared using the Chi-square test and Fisher’s exact test. Survival analysis was performed using Kaplan-Meier curves and differences were assessed with the log-rank test. Differences were considered significant at *P* < 0.05.

## Results

### General data analysis

Analysis of epidemiological data revealed a lower average age and proportion of menopause of patients in the EAOC than non-EAOC group (*P* < 0.05). No significant differences in BMI, dysmenorrhea history, menopausal age, endometriosis history, operation history, and complications were observed between the two groups (all *P* > 0.05), as shown in Table 1.

**Table 1:** Comparative analysis of the epidemiological data of EAOC and non-EAOC patients (n, x ± s)

Characteristics	EAOC	Non-EAOC	P
	74	100	
Age(year)	49.4 ± 7.7	54.0 ± 10.0	**0.001**
BMI	23.6 ± 3.3	23.5 ± 3.7	0.731
History of dysmenorrhea			0.064
Yes	29	26	
No	55	74	
Menopausal status			**0.001**
Yes	32	68	
No	42	32	
Age of menopause	49.1 ± 4.3	49.3 ± 3.9	0.815
Endometriosis history			0.980
Yes	11	15	
No	63	85	
History of surgery			0.115
Yes	19	16	
Caesarean section	11	6	
Hysterectomy	4	7	
Endometriosis	4	3	
No	65	84	
Complications			0.683
Yes	31	45	
No	43	55	

Further intra-group and inter-group analyses of the same pathological types showed a significantly lower average age of onset and proportion of menopause in the EOCC than OCC group (*P* < 0.05). We observed no marked differences in the remaining parameters between the groups (*P* > 0.05; Table 2).

**Table 2:** Intra-group and inter-group analyses of EAOC and non-EAOC patients (n, x ± s)

Characteristics	EOEC	EOCC	OEC	OCC	P
	35	39	65	35	
Age(year)	50.6 ± 8.4	48.2 ± 6.7	54.3 ± 10.7	53.3 ± 8.6	0.177^a^/0.060^b^/**<0.01** ^c^
BMI	23.9 ± 2.9	23.3 ± 3.6	23.7 ± 3.8	22.8 ± 3.1	0.430^a^/0.782^b^/0.570^c^
History of dysmenorrhea					0.076^a^/0.667^b^/0.075^c^
Yes	10	19	16	10	
No	25	20	49	25	
Postmenopausal status					0.178^a^/0.199^b^/**<0.01** ^c^
Yes	18	14	42	26	
No	17	25	23	9	
Age of menopause	48.8 ± 4.7	49.2 ± 3.8	49.9 ± 4.1	48.2 ± 3.2	0.802^a^/0.408^b^/0.363^c^
Endometriosis history					0.894^a^/0.883^b^/0.894^c^
Yes	5	6	10	5	
No	30	33	55	30	
History of surgery					0.599^a^/0.106^b^/0.810^c^
Yes	8	11	7	9	
Caesarean section	3	8	2	4	0.149^a^/0.340^b^/0.290^c^
Hysterectomy	3	1	3	4	0.339^a^/0.420^b^/0.183^c^
Endometriosis	2	2	2	1	0.911^a^/0.610^b^/0.475^c^
No	27	28	58	26	
Complications					0.528^a^/0.916^b^/0.892^c^
Yes	16	15	29	14	
No	19	24	36	21	

^a^:EOEC vs. EOCC, ^b^:EOEC vs. OEC, ^c^:EOCC vs. OCC

### Analysis of clinical features

No significant differences were evident between the two groups in terms of tumor size, tumor side, ascites, type of surgery, CA125, HE4 and CA19.9 levels (all *P* > 0.05). The collective data are presented in Table 3.

**Table 3:** Comparative analysis of clinical features of EAOC and non-EAOC patients (n, x ± s)

Characteristics	EAOC	Non-EAOC	P
	74	100	
Ascites			0.666
Yes	39	56	
No	35	44	
Tumor size			0.769
≥ 10 cm	45	63	
<10 cm	29	37	
Tumor side			0.355
Unilateral	53	65	
Bilateral	21	35	
Type of surgery			0.804
R0	66	87	
R1	9	10	
R2	2	3	
Biomaker			
CA125(U/ml)	448.9 ± 980.0	739.8 ± 1141.4	0.080
HE4(pmol/L)	203.4 ± 240.0	264.5 ± 217.5	0.620
CA19.9(U/ml)	194.0 ± 460.4	274.2 ± 546.7	0.465

R0: no residual lesions; R1: residual lesions < 1 cm; R2: residual lesions > 1 cm

Further intra-group and inter-group analyses disclosed significantly higher incidence of bilateral tumors in the EOEC than EOCC group (40.0% vs. 17.9%, *P* < 0.05). Moreover, the HE4 level in the EOEC group was higher than that in the EOCC group to a significant extent (355.4 vs. 89.4 pmol/L, *P* < 0.05). No significant differences were found in the remaining parameters, including tumor size, tumor side, ascites and type of surgery (Table 4).

**Table 4:** Intra-group and inter-group clinical features analyses of EAOC and non-EAOC patients (n, x ± s)

Characteristics	EOEC	EOCC	OEC	OCC	P
	35	39	65	35	
Ascites					0.469^a^/0.295^b^/0.209^c^
Yes	20	19	44	12	
No	15	20	21	23	
Tumor size					0.892^a^/0.881^b^/0.363^c^
≥ 10 cm	21	24	38	25	
<10 cm	14	15	27	10	
Tumor side					**0.036** ^a^/0.303^b^/0.316^c^
Unilateral	21	32	33	32	
Bilateral	14	7	32	3	
Type of surgery					0.894^a^/0.883^b^/0.590^c^
R0	30	33	55	32	
R1	3	6	8	2	
R2	2	0	2	1	
Biomaker					
CA125(U/ml)	618.0 ± 1309.8	297.0 ± 509.6	870.3 ± 895.0	493.7 ± 1479.0	0.161^a^/0.255^b^/0.437^c^
HE4(pmol/L)	355.4 ± 319.5	89.4 ± 69.1	498.9 ± 547.0	132.7 ± 259.0	**0.039** ^a^/0.575^b^/0.651^c^
CA19.9(U/ml)	248.1 ± 525.4	160.6 ± 423.0	276.9 ± 586.0	220.5 ± 547.0	0.556^a^/0.877^b^/0.663^c^

^a^:EOEC vs. EOCC, ^b^:EOEC vs. OEC, ^c^:EOCC vs. OCC.

R0: no residual lesions; R1: residual lesions < 1 cm; R2: residual lesions > 1 cm

### Pathological characteristics

**Fig. 1:**
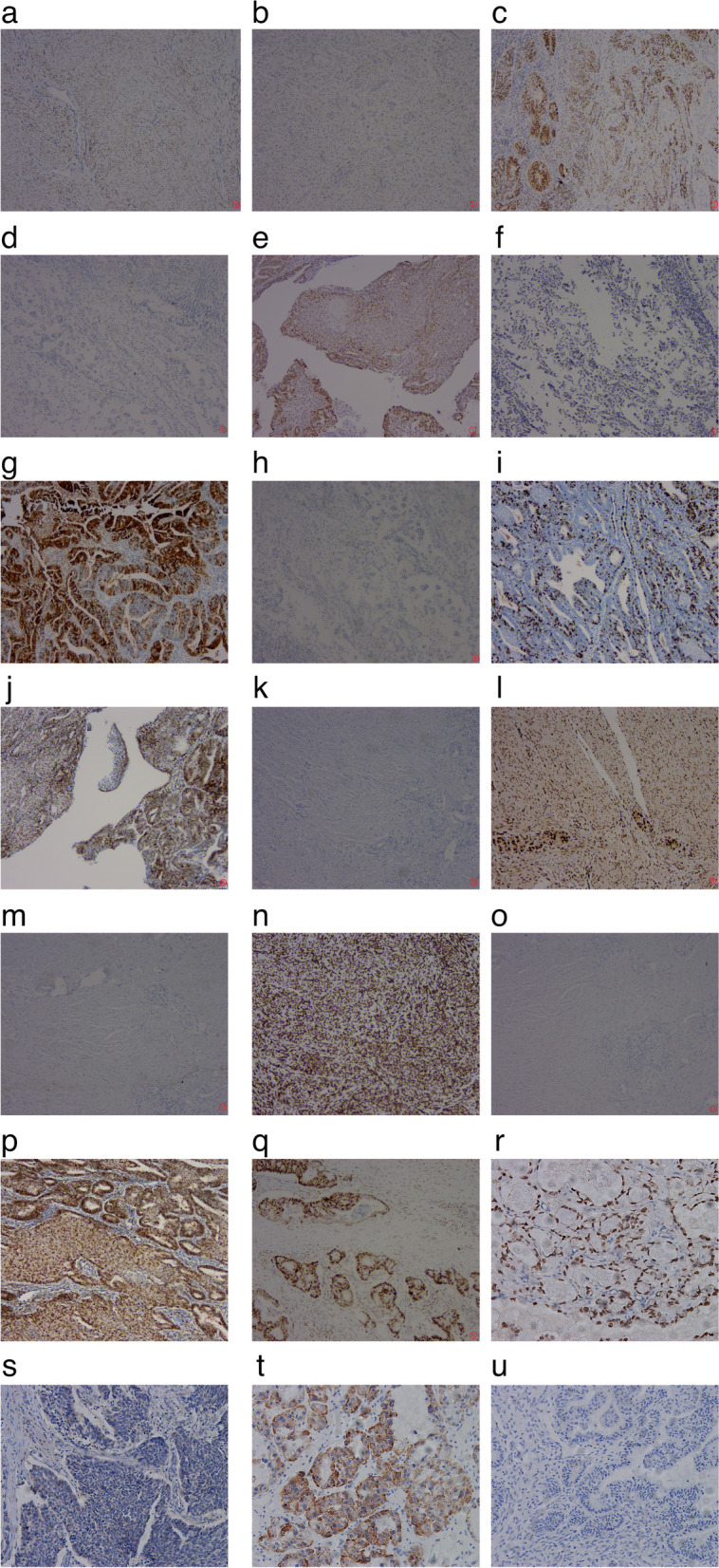
IHC expression of related molecules in ovarian tissues (SP 200X). **a** ER-positive; **b** ER-negative; **c** PR-positive; **d** PR-negative; **e** P53-positive; **f** P53-negative; **g** P16-positive; **h** P16-negative; **i**. Ki67-positive; **j** MLH1-positive; **k** MLH1-negative; **l**. MSH2-positive; **m** MSH2-negative; **n** MSH6-positive; **o** MSH6-negative; **p** PMS2-positive; **q** PMS2-negative; **r** HNF-1β-positive; **s** HNF-1β-negative; **t** Napsin A-positive; **u** Napsin A-negative

Pathological characteristics were comparable between the two groups in terms of FIGO stage, differentiation degree, ER, PR, P53, P16, Ki67, MMR, HNF-1, and Napsin A-positive expression (all *P* > 0.05; Table 5; Fig. 1).

**Table 5:** Comparative analysis of pathological characteristics of EAOC and non-EAOC patients(n, %)

Characteristics	EAOC	Non-EAOC	P
	74	100	
FIGO stage			0.417
I/II	51	63	
III/IV	23	37	
Differentiation			0.932
Low	18	21	
Middle	34	52	
High	22	27	
Biomarker			
ER(+)	32	52	0.253
PR(+)	24	45	0.094
P53(+)	59	68	0.085
P16(+)	41	54	0.854
MMR(-)	19	37	0.114
Ki67(%)	30.0 ± 18.0	30.8 ± 18.94	0.767
Napsin A(+)	18	37	0.075
HNF-1β(+)	15	31	0.113

The rates of ER and PR positivity in the EOEC group were significantly higher than those in the EOCC group (71.4% vs. 17.9%, 60.0% vs. 7.7%; *P* < 0.05). Conversely, the rates of HNF-1 and Napsin A positivity in the EOCC group were markedly higher relative to the EOEC group (28.2% vs. 20.0%, 30.8% vs. 7.7%; *P* < 0.05).

HNF-1- and Napsin A-positive rates in the OCC group were significantly higher than those in the OEC group (74.2% vs. 16.9%, 80.0% vs. 4.6%; *P* < 0.05), as shown in Table 6.

**Table 6:** Intra-group and inter-group pathological characteristics of EAOC and non-EAOC patients(n, %)

Characteristics	EOEC	EOCC	OEC	OCC	P
	35	39	65	35	
FIGO stage					
I/II	21	30	37	26	0.116^a^/0.767^b^/0.792^c^
III/IV	14	9	28	9	
Differentiation					
Low	11	7	18	3	0.177^a^/0.695^b^/0.239^c^
Middle	17	17	40	12	
High	7	15	7	20	
Biomarker					
ER(+)	25	7	49	3	**< 0.01** ^a^/0.667^b^/0.311^c^
PR(+)	21	3	40	5	**< 0.01** ^a^/0.880^b^/0.635^c^
P53(+)	29	30	43	25	0.526^a^/0.076^b^/0.589^c^
P16(+)	16	25	32	22	0.112^a^/0.710^b^/0.912^c^
MMR(-)	8	11	22	15	0.072^a^/0.548^b^/0.291^c^
Ki67(%)	29.1 ± 20	30.7 ± 14	29.6 ± 19	33.0 ± 16.5	0.718^a^/0.907^b^/0.564^c^
Napsin A(+)	7	11	11	26	**< 0.01** ^a^/0.459^b^/0.230^c^
HNF-1β(+)	3	12	3	28	**< 0.01** ^a^/0.317^b^/0.273^c^

^a^:EOEC vs. EOCC, ^b^:EOEC vs. OEC, ^c^:EOCC vs. OCC

### Prognostic characteristics

Overall, 74 patients in the EAOC group were subjected to initial analyses, 64 of whom were followed up (follow-up rate of 86.5%). Rates of platinum therapy resistance, recurrence and mortality were determined as 6.25%, 39.1%, and 28.1%, respectively. Among the 100 patients in the non-EAOC group, 85 were followed up (85.0%). The platinum resistance rate was determined as 9.41%, recurrence rate as 54.1%, and mortality rate as 40.0%, which were not significantly different between the two groups (all *P* > 0.05; Table 7).

**Table 7:** Prognosis comparison between EAOC and non-EAOC patients(n)

Characteristics	EAOC	non-EAOC	P
	74	100	
**Platinum resistance**	4	8	0.483
**Relapse**	25	39	0.405
**Death**	19	34	0.193

**Fig. 2:**
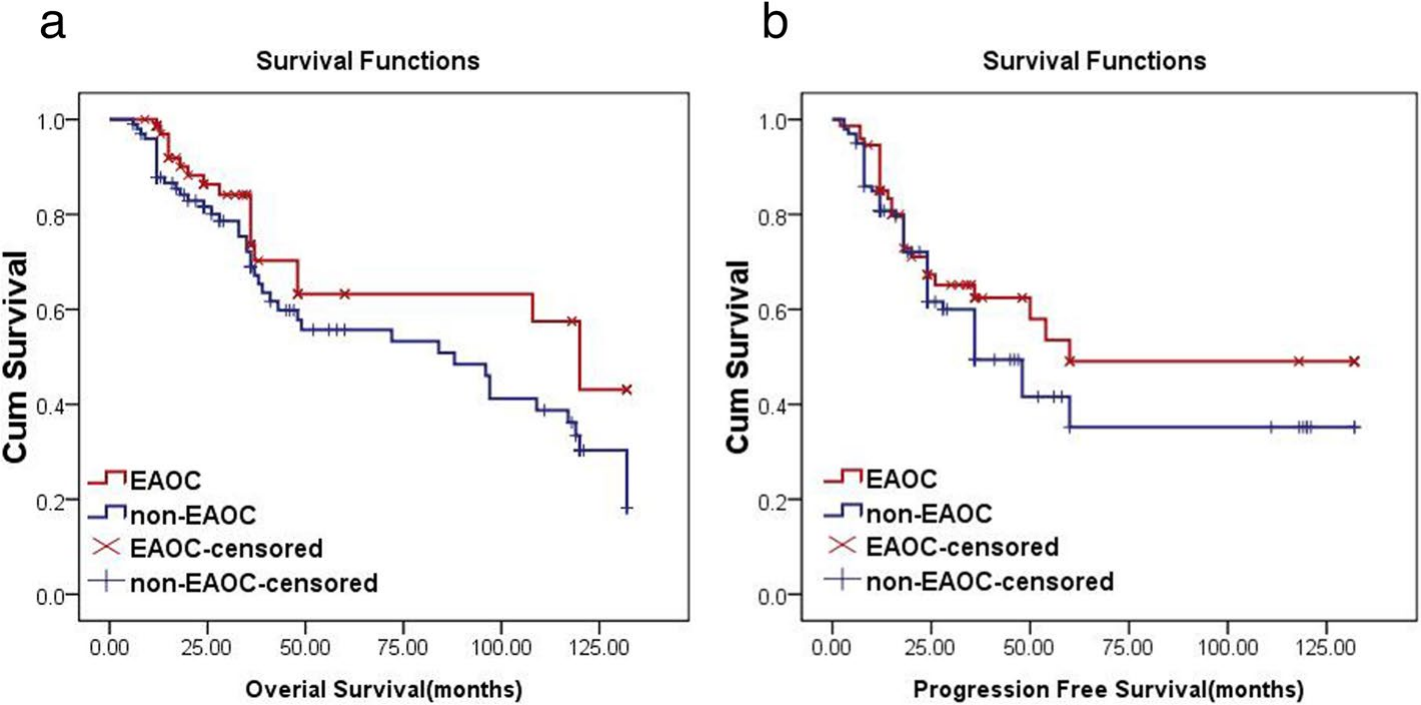
Differences in OS and PFS between EAOC and non-EAOC patients. **a** OS; **b** PFS

Kaplan-Meier analysis and log-rank test showed that the average overall survival (OS) of the EAOC group was 91.6 months (95% CI: 76.9–106.5 months) while that of the non-EAOC group was 77.8 months (95% CI: 66.1–90.0 months), with no significant differences (*P* = 0.068, > 0.05). The median progression-free survival (PFS) of the EAOC group was 78.4 months (95% CI: 62.2–94.5 months), which was not significantly different from the non-EAOC group (64.0 months; 95% CI: 50.7–77.1 months) (*P* = 0.216, > 0.05), as shown in Fig. 2.

**Fig. 3:**
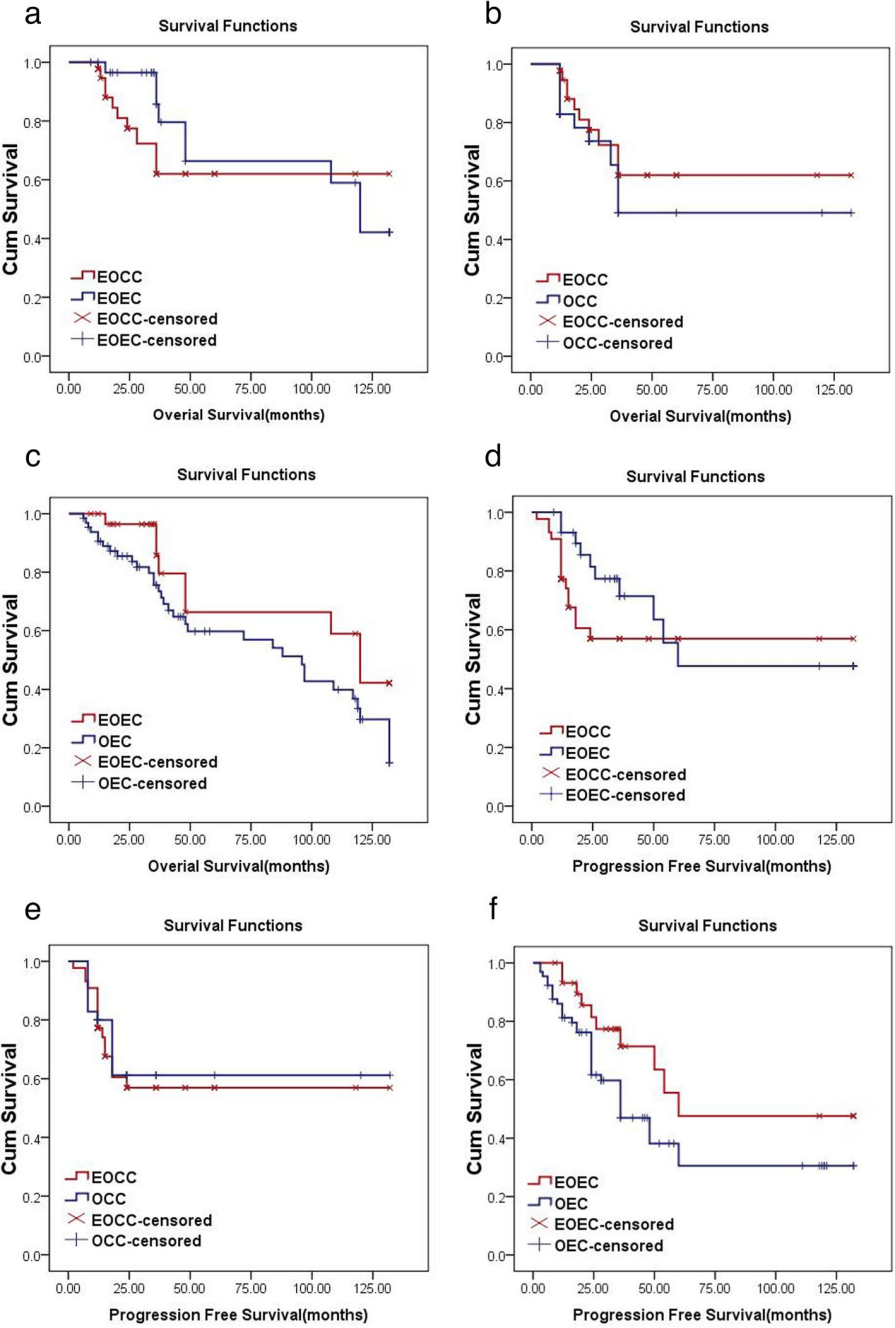
Differences in OS and PFS of EOCC *vs. *EOEC, EOCC *vs. *OCC and EOEC *vs. *OEC groups. **a** OS-EOCC *vs. *EOEC; **b** OS- EOCC *vs. *OCC; **c** OS- EOEC *vs. *OEC; **d** PFS- EOCC *vs. *EOEC; **e** PFS- EOCC *vs. *OCC; **f** PFS- EOEC *vs. *OEC

Intra-group and inter-group analyses of the same pathological types via Kaplan-Meier and log-rank tests showed that OS of the EOCC group was 90.8 months (95% CI, 69.9–111.8 months), EOEC group was 96.8 months (95% CI, 76.8–116.8 months), OCC group was 77.4 months (95% CI, 51.49–103.30 months), and OEC group was 81.10 months (95% CI, 68.0–94.2 months). We observed no significant differences in OS in EOCC vs. EOEC, EOCC vs. OCC, and EOEC vs. OEC groups (*P* = 0.290, 0.262, 0.070, all *P* > 0.05). PFS of patients in the EOCC group was 80.9 months (95% CI, 61.2–100.6 months), EOEC group was 82.3 months (95% CI, 59.4–105.2 months), OCC group was 85.9 months (95% CI, 68.4–108.3 months), and OEC group was 60.0 months (95% CI, 45.50–74.6 months). We observed no significant differences in PFS in EOCC vs. EOEC, EOCC vs. OCC, and EOEC vs. OEC groups (*P* = 0.222, 0.675, 0.071, all *P* > 0.05; Fig. 3).

## Discussion

Several recent studies have highlighted a significantly increased risk of ovarian cancer in endometriosis patients [12]. As a precancerous lesion, endometriosis is closely related to ovarian clear cell and endometrioid carcinomas. The theory of external origin of ovarian cancer hypothesizes that both clear cell and endometrioid carcinoma of the ovary originate from malignant transformation of endometriosis, which poses a challenge to the current diagnostic criteria for endometriosis-associated malignant transformation. However, further clinical and basic research evidence is needed to substantiate this theory. In the current study, the two ovarian cancer types (clear cell and endometrioid carcinomas) most closely associated with endometriosis were examined as a whole. According to the diagnostic criteria of Sampson and Scott, samples were divided into EAOC and non-EAOC groups for comparison of clinicopathologic features and prognosis. Our results showed no significant differences between the groups, supporting the theory that both non-endometriosis-associated primary ovarian endometrial carcinoma and ovarian clear cell carcinoma potentially have the same origin as EAOC from endometriosis.

We retrospectively analyzed the clinical features of 174 patients from EAOC and non-EAOC groups. The average age and proportion of menopausal patients in the EAOC group was lower relative to the non-EAOC group (*P* < 0.05). These differences may be attributed to a potential decrease in postmenopausal hormone levels, gradual atrophic degradation of ectopic endometrium [13], limitations of pathological materials, and errors due to insufficient pathological evidence of endometriosis. Further experiments on larger sample sizes are required to confirm these findings. No significant differences were evident in BMI, dysmenorrhea history, endometriosis history, operation history, complications, ascites, tumor size, type of surgery and relevant serum tumor markers (CA125, HE4, CA19.9) between the two groups (all *P* > 0.05), consistent with earlier results. Our experiments support similar characteristics of endometriosis- and non-endometriosis-associated primary ovarian cancers. Furthermore, upon stratified analysis according to pathological type into EOCC and EOEC, OCC and OEC groups, relevant epidemiological and clinical characteristics, such as BMI, dysmenorrhea history, endometriosis history, history of surgery, complications, ascites, tumor size, type of surgery and serum tumor marker (CA125, HE4, CA19.9) levels, were not significantly different (*P* > 0.05). Our findings present further evidence that primary ovarian endometrial carcinoma and clear cell carcinoma are associated with corresponding pathological types in endometriosis-associated ovarian cancer with similar clinical features. These results provide preliminary confirmation that the two groups have comparable epidemiological and clinical characteristics and the age difference is potentially attributed to strict diagnostic criteria.

Histopathological evaluation is currently the gold standard of ovarian cancer diagnosis and classification. Immunohistochemical analysis revealed high expression of ER and PR in endometrioid carcinoma of ovary and low expression in clear cell carcinoma [14]. Compared with other epithelial ovarian cancer types, increased HNF-1β, high expression of Napsin A and positivity for P53 were characteristic features of ovarian clear cell carcinoma [15]. MMR expression in both ovarian endometrial and clear cell carcinoma types is abnormally high relative to that in other subtypes of ovarian cancer [16]. The above molecules may therefore serve as useful biomarkers to distinguish epithelial ovarian cancer subtypes. Ki-67 is currently used as a positive nuclear proliferation marker and its expression reflects the biological behavior of tumor cells [17]. For analysis of differences, similarities and potential mechanisms of EAOC and non-EAOC, we compared the pathologies of the two groups. Our data showed no histological differences in terms of pathologic differentiation degree, ER, PR, P53, and P16, Ki67, MMR, HNF-1β and Napsin A-positive expression (*P* > 0.05) between EAOC and non-EAOC. The majority of previous reports have investigated the malignant transformation of endometriosis, with limited studies focusing on the characteristics of different pathological types of endometrial carcinoma and clear cell carcinoma. However, a number of differences in clinicopathological characteristics exist between endometrioid and clear cell carcinoma types. Further grouping analysis revealed no significant differences in expression of relevant non-specific indicators, such as P53, P16, Ki67 and MMR, among the groups. The positive expression rates of ER and PR were markedly different between endometrioid carcinoma and clear cell carcinoma groups, but not from the primary ovarian carcinoma of the same pathologic type. Differentiation of endometriosis into two tissue types is reported to occur in a dual mode regulated by sex hormones [18, 19]: (1) estrogen and progesterone receptor-positive endometriosis lesions undergo malignant transformation to form hormone-dependent endometrioid carcinoma after long-term stimulation without antagonistic estrogen and (2) atrophic ectopic endometrial lesions negative for estrogen and progesterone receptors are stimulated by oxidative stress for a long period of time, resulting in malignant transformation and formation of non-hormone-dependent clear cell carcinoma. Our results were consistent with earlier literature, validating that clear cell carcinoma is a non-hormone-dependent tumor. The positive expression rates for HNF-1β and Napsin A were significantly higher in clear cell carcinoma than endometrioid carcinoma. Accordingly, we concluded that different histological types undergo distinct mechanisms for development of endometriosis-associated ovarian cancer, which will be the focus of follow-up investigations. The above results further confirm similar expression of pathology-related molecular indicators in EAOC and non-EAOC with the same pathological subtype. Moreover, the differences in molecular expression indicated by the results of stratified analysis based on pathological subtype may be related to the pathogenesis of the different types.

The majority of previous studies focused on comparing clinicopathological and prognostic differences between intrauterine endometriosis-associated ovarian cancers of a single pathologic type. However, sample sizes in earlier reports were usually small and the results obtained were inconsistent. Some studies suggest that ovarian endometrial carcinoma and clear cell carcinoma are early phases with better prognosis than the two pathological types of primary ovarian cancer [20, 21], while other researchers report no obvious differences [22, 23]. Here, we examined 174 cases of ovarian endometrial carcinoma and clear cell carcinoma, with a median follow-up period of 67 months. Our data showed no significant differences between platinum resistance, recurrence and mortality rates between the EAOC and non-EAOC groups (*P* > 0.05). PFS and OS were comparable between the two groups (*P* > 0.05), supporting similar prognosis. In 2020, Hermens et al. [24] analyzed 32,419 patients with ovarian cancer and found synchronous endometriosis of ovarian cancer staging of early, higher progression-free survival and overall survival is longer, perhaps because of more frequent hospital visits of patients with endometriosis due to simultaneous treatment with the appropriate drugs and long-term state of endometriosis-induced inflammation, which activates immune function, in turn, facilitating early detection and better prognosis, giving rise to the theory that ovarian cancer with endometriosis may have different pathophysiological features relative to other ovarian cancer types. Earlier studies have established a low early incidence of epithelial ovarian cancer. In this research, the early incidence rates of EAOC and non-EAOC were 68.9% and 63.0%, respectively, which were significantly higher than the average early incidence of ovarian cancer (30.0%). Overall survival rates of the EAOC and non-EAOC groups were 71.9% and 60.0%, which were markedly higher than the average five-year survival rate of ovarian epithelial carcinoma (44- 50%) [25]. Considerable evidence suggests that ovarian clear cell carcinoma and endometrioid carcinoma are sources of malignant transformation of endometriosis and can therefore be detected earlier. However, accelerated growth of tumor tissue destroys the tissue of origin owing to malignant transformation. Pathological sampling cannot remove cancer and ectopic foci lesions at the same time. Consequently, a proportion of clear cell and endometrioid carcinomas of the ovary are not diagnosed as endometriosis-associated ovarian cancer. Our results indicate similar rates of recurrence and survival in EAOC and non-EAOC groups.

In summary, this study found no significant differences in epidemiological or pathological features and comparable prognosis between endometriosis- and non-endometriosis-associated ovarian cancer diagnosed according to the Sampson and Scott criteria, leading to the speculation that primary ovarian endometrial and clear cell carcinomas have the same origin as EAOC. Our findings support the theory of dualism of ovarian cancer suggesting that both endometrial carcinoma and clear cell carcinoma originate from progression of endometriosis. Based on our findings, we propose that the Sampson and Scott diagnostic criteria for endometriosis malignancy are too strict and the actual incidence of malignant transformation of endometriosis may be higher. Therefore, it is worth exploring whether the stringent diagnostic criteria for malignant transformation of endometriosis require further revision. In view of the collective data, the diagnostic criteria for endometriosis malignancy could include the following guidelines: (1) no other primary tumor site must exist, (2) the histology of the tumor should be consistent with an endometrial origin, and exclude: there must be a clear example of endometriosis in association with or close proximity to the cancer and endometriosis associated with cancers must show morphologic progression from benign to malignant in a contiguous manner.

The retrospective nature of this study is a major limitation. Moreover, the results represent single-center data and the number of included cases is relatively low. Further large-scale, prospective multicenter clinical and molecular biology studies are therefore required to validate the associations of malignant transformation of endometriosis with ovarian clear cell and endometrial carcinomas, which should aid in clarification of the underlying biological mechanisms and development of individualized treatments for patients with endometriosis-associated ovarian cancer.

## Data Availability

The datasets generated and/or analyzed in the current study are not publicly available due to the data also forming part of an ongoing study, but can be made available by the corresponding author on reasonable request.

## Abbreviations

EAOC: Endometriosis-associated ovarian cancer
non-EAOC: Non-endometriosis-associated primary ovarian cancer
EOCC: Endometriosis associated ovarian clear cell carcinoma
EOEC: Endometriosis associated ovarian endometrioid carcinoma
OCC: Clear cell carcinoma
OEC: Endometrioid carcinoma
BMI: Gody Mass Index
OS: Overall survival
PFS: Progression-free survival
